# State Microscopical Society of Illinois

**Published:** 1880-06

**Authors:** 


### ARTICLE VII.

### STATE MICROSCOPICAL SOCIETY OF ILLINOIS.

The annual meeting of the State Microscopical Society of Illinois was held at the Academy of Sciences, 263 Wabash avenue, Friday evening, April 23, 1880.

The treasurer's report showed a highly satisfactory financial condition, about $200 having been paid in during the past year, while the expenditures were less than $50.

The following papers have been read before the society since the semi-annual meeting, last October:

Recent Microscopical Work, by James Colgrove.

The Microscopical Examination of Signatures, by Lester Curtis.

The Microscopical Examination of Dust, by A. C. Thomas.

A New Observation on the Histology of the Fœtal Lung, by Lester Curtis.

The Microscopical Examination of Tissues after the Administration of Mercury, by S. V. Clevenger.

The Study of the Cell, with Reference to the New Theory, by Lester Curtis.

Plant or Animal? A Popular Description of some of the Myogastric Fungi, by the Secretary.

Notes on Micro-lithology, by A. C. Clark.

The Intra-ovular Life of the Chick, by C. H. Kimball.

The following gentlemen were elected: President, B. W. Thomas; Vice-Presidents, Lester Curtis, M.D., Prof. E. J. Bastin; Secretary, E. B. Stuart; Corresponding Secretary, James

Colgrove; Treasurer, W. H. Summers; Trustees, Prof. E. J. Hill, Dr. S. J. Jones, Dr. F W Mercer, H. M. Thompson and Chas. Boring. E. B. STUART, *Secy.*

---

At a meeting of regular physicians of Aurora and vicinity, held at Aurora, Ill., March 15, 1880, a medical society was organized and named the Aurora Medical Society. It is designed to be auxiliary to the Illinois State Medical Society and to meet on the second Monday of January, March, May, July, September and November. The officers for the present year are as follows: President, A. Hard, M.D. of Aurora, Ill.; Vice President, D. S. Jenks, M.D. of Plano, Ill.; Secretary and Treasurer, M. M. Robbins, M.D. of Aurora, Ill.

---

HYDRATE OF CHLORAL AND OXIDE OF ZINC IN ACUTE INTESTINAL AFFECTIONS OF CHILDREN.—(*Archiv. Clin. Ital.*, No. 3, 1880.)

Dr. James Tison has found useful a combination of hydrate of chloral and oxide of zinc in cases of intestinal irritation, infantile diarrhœa, summer diarrhœa, diarrhœa of nurslings, etc. He gives the chloral in clysters and the oxide of zinc by the mouth. At the same time a strict dietetic regime is enforced. A solution of a (℈j)gram and a half of chloral in 60 grams of starch water is made, of which a teaspoonful to a teaspoonful and a half is injected two or three times a day. In addition to this, a teaspoonful of the following mixture is given every five hours:

| | | | |
|---|---|---|---|
| Oxide of zinc | (℈j) | 1 | 50 |
| Gum Arabic, Sugar āā | (ʒij) | 7 | 50 |
| Lactopeptine | (ʒj) | 3 | 50 |
| Cannella water | (℥j) | 32 | |

